# Perception towards vaccine effectiveness in controlling COVID-19 spread in rural and urban communities: A global survey

**DOI:** 10.3389/fpubh.2022.958668

**Published:** 2022-09-26

**Authors:** Roy R. Marzo, Rajeev Shrestha, Binaya Sapkota, Swosti Acharya, Nita Shrestha, Mandip Pokharel, Absar Ahmad, Mark E. Patalinghug, Farzana Rahman, Zahir R. Salim, Burcu K. Bicer, Masoud Lotfizadeh, Baniissa Wegdan, Edlaine F. de Moura Villela, Kittisak Jermsittiparsert, Nouran A. Hamza, Marina R. Saleeb, Titik Respati, Susan Fitriyana, Sudip Bhattacharya, Petra Heidler, Sikandar A. Qalati, Yadanar Aung, Khadijah Abid, Tayachew A. Abeje, Ashmita Pokhrel, Rohullah Roien, Isabel King, Tin Tin Su

**Affiliations:** ^1^Department of Community Medicine, International Medical School, Management and Science University, Shah Alam, Malaysia; ^2^Global Public Health, Jeffrey Cheah School of Medicine and Health Sciences, Monash University Malaysia, Subang Jaya, Malaysia; ^3^Department of Public Health, Faculty of Medicine, Asia Metropolitan University, Masai, Malaysia; ^4^Department of Pharmacy, District Hospital Lamjung, Besisahar, Nepal; ^5^Department of Pharmaceutical Sciences, Nobel College, Affiliated to Pokhara University, Kathmandu, Nepal; ^6^Nepal Health Research and Innovation Foundation, Kathmandu, Nepal; ^7^Vennue Foundation, Kathmandu, Nepal; ^8^Department of Community Medicine, Manipal Tata Medical College, Manipal Academy of Higher Education, Jamshedpur, India; ^9^School of Criminal Justice Education, J.H. Cerilles State College, Zamboanga, Philippines; ^10^Administration and Research, Bangladesh National Nutrition Council, Ministry of Health & Family Welfare, Dhaka, Bangladesh; ^11^College of Business Administration, International University of Business Agriculture and Technology, Dhaka, Bangladesh; ^12^Department of Medical Education and Informatics, Faculty of Medicine, Gazi University, Ankara, Turkey; ^13^Department of Community Health, Shahrekord University of Medical Sciences, Shahr-e Kord, Iran; ^14^College of Health Sciences/Nursing Department, Sharjah Institute of Medical and Health Sciences, University of Sharjah, Sharjah, United Arab Emirates; ^15^Public Policies, Education and Communication, Disease Control Coordination, São Paulo State Health Department, São Paulo, Brazil; ^16^Faculty of Administrative, Economic and Social Sciences, University of City Island, Northern Cyprus, Turkey; ^17^Medical Agency for Research and Statistics, Giza, Egypt; ^18^Clinical Research Key, Nairobi, Kenya; ^19^Department of Biostatistics, Medical Agency for Research and Statistics, Giza, Egypt; ^20^Faculty of Medicine, Universitas Islam Bandung, Bandung, Indonesia; ^21^All India Institute of Medical Sciences, Deoghar, India; ^22^Department for Economy and Health, Faculty of Health and Medicine, University for Continuing Education Danube University Krems, Krems, Austria; ^23^Department of Health Sciences, St. Pölten University of Applied Sciences, St. Pölten, Austria; ^24^Department of International Business and Export Management, IMC University of Applied Sciences Krems, Krems, Austria; ^25^School of Finance and Economics, Jiangsu University, Zhenjiang, China; ^26^Medical Statistics Division, Department of Medical Research, Ministry of Health, Myanmar, Myanmar; ^27^Department of Public Health, Shaheed Zulfiqar Ali Bhutto Institute of Science and Technology, Karachi, Pakistan; ^28^Department of Biology MSc. in Genetics, Mizan Tepi University, Tepi, Ethiopia; ^29^Department of Nursing, Lumbini Medical College & Teaching Hospital, Tansen, Nepal; ^30^Medical Research Center, Kateb University, Kabul, Afghanistan; ^31^Department of Exercise Physiology, School of Health and Behavioral Sciences, University of the Sunshine Coast, Sunshine Coast, QLD, Australia; ^32^South East Asia Community Observatory (SEACO), Monash University Malaysia, Johor, Malaysia

**Keywords:** COVID-19, global study, predictors, vaccine acceptance, perception towards vaccine effectiveness

## Abstract

**Introduction:**

Several studies exhibited varying reports of perception toward vaccine effectiveness, vaccine hesitancy, and acceptance of COVID-19 vaccines. As this fluctuated with evidence generation, this study explored the perception toward vaccine effectiveness in rural and urban communities among various countries.

**Methods:**

A cross-sectional study was conducted online from April to August 2021 using convenience sampling among people from different countries approved by the Asia Metropolitan University Medical Research and Ethics. We adapted the questionnaire from the World Health Organization's (WHO) survey tool and guidance on COVID-19. The logistic regression models were performed to show perception toward vaccine effectiveness.

**Results:**

A total of 5,673 participants responded to the online survey. Overall, 64% of participants agreed that the vaccine effectively controlled viral spread, and 23% agreed that there was no need for vaccination if others were vaccinated. Males had 14% higher odds of believing that there was no need for vaccination. Less social media users had 39% higher odds of developing the belief that there is no need for vaccination than all other people vaccinated.

**Conclusion:**

People's perceptions toward vaccine acceptance have fluctuated with the information flow in various social media and the severity of COVID-19 cases. Therefore, it is important that the current scenario of peoples' perception toward vaccine acceptance and determinants affecting the acceptance are explored to promote the vaccination approach against COVID-19 prevention and transmission effectively.

## Introduction

The spread of coronavirus disease 2019 (COVID-19) has affected the worldwide (1–3). Although vaccines may not fully protect from the COVID-19, it is one of the most important public health interventions as the full range of vaccination among community people can help protect from transmission of infection from the infected to the uninfected and control potential death (4–9). While herd immunity achieved with vaccination is a potential public health intervention against COVID-19, vaccine hesitancy (i.e., reluctance in vaccine acceptance or even delays in refusal amidst the availability of safety- and effectiveness-assured vaccination facilities) has become a global public health concern (4–10). COVID-19 vaccine acceptance or hesitancy, like in the case of other vaccines, is context-specific, varying across the country, time, and place (8) due to socio-demographic differences, health conditions, individual cognitive, psychological and behavioral factors, awareness about vaccines' safety, effectiveness and potential side effects, fast development compared to other vaccines, perceived lack of testing, control of myths, confidence in the health system, and political and cultural factors. Since vaccine hesitancy plays a significant barrier to successful vaccination campaigns, the availability of COVID-19 vaccines does not solve the issue (4, 7, 11–13). Also, Covid vaccine hesitancy reflected an interesting public perception that it rose significantly when new and deadly variants emerged (14). Hence, health workers and policymakers should address the root cause of hesitancy to successfully make the global vaccine action plan (11, 13). The SAGE Working Group on Vaccine Hesitancy concluded that vaccine hesitancy refers to “*delay in acceptance or refusal of vaccination despite availability of vaccination services. Vaccine hesitancy is complex and context-specific, varying across time, place, and vaccines.”* Vaccine hesitancy is influenced by factors such as complacency, convenience, and confidence (15). Vaccine hesitancy is usually guided by three major factors: individuals' perception toward all vaccination programs, including COVID-19 vaccine peers' influence, and perceived behavioral control (7).

Some people may initially show hesitancy due to less awareness about vaccination, cost implications, and poor or substandard health literacy, but later may be interested after they become aware of the long-term safety data with vaccination (13, 16). A case in point was that 91% were willing to get the COVID vaccine in Ecuador, if it is at least 95% effective (17). Vaccine hesitancy is especially problematic for individuals with chronic diseases, disabilities, those requiring long-term care facilities, and geriatric patients (18). The anti-vax groups' conspiracy theories, misperceptions, and expert opinions on the consequences of the COVID-19 vaccine are also fueling hesitancy (16). In India, a massive mass of target users usually shows vaccine hesitancy even for routine immunization, which was reflected in the hesitancy to measles-rubella vaccine in 2016 (5), which was previously reported in the USA (16). Different studies have exhibited varying reports of hesitancy and acceptance of COVID-19 vaccines (9, 19, 20). As this fluctuated with evidence generation, this study explored the perception toward vaccine effectiveness in rural and urban communities among various countries. The study findings would help the policymakers and practitioners become aware of the latest trends and determinants in the success of vaccination and devise efficient and effective strategies for the same.

## Methods

### Study design and sampling

A cross-sectional online survey was conducted online from April to August 2021 using convenience sampling among people from five different countries. Bangladesh, Iran, Malaysia, Philippines, and Turkey were selected for the study based on investigation resources within our existing international research group and high disease burden of COVID-19. The sample sizes for each country were calculated *n* = 384 according to sample size calculation using 95% CI, 50% response, and 0.05 margin of error (21). The study was conducted using convenience sampling *via* web-based online method. According to Stratton, the convenience sampling participants are available around a location, Internet site, or customer-membership list. It is an acknowledged form of sampling and is often found in population research and disaster research (22). The questionnaires were shared to be filled by participants from April to August 2021. The response received during that period was cleared and taken into analysis.

### Ethics approval

The study was approved by the Asia Metropolitan University Medical Research and Ethics (Ref. AMU FOM 0400132021).

### Instrument development and measures

The questionnaire was adapted from the World Health Organization's (WHO) survey tool and guidance on COVID-19 (23). All participants were informed about the survey's purpose and provided their informed consent before participation. Participants were ensured of the confidentiality and privacy of their responses to reduce potential bias introduced by self-reported data. The participants could only complete the questionnaires once, and the Google form was set to receive anonymous responses without identifying emails or contact details. The questionnaire was structured into two sections: (1) socio-demographic characteristics and medical history and (2) perception of COVID-19 vaccine effectiveness.

The questionnaire was initially developed in English and translated into local languages. Then, the research team back-translated, pre-tested, and revised the questionnaire in the selected five countries. A group of expert panels in the respective countries included psychiatrists, clinical psychologists, physicians, and public health experts translated and culturally validated into their national. Pilot testing comprised 15 participants in each country to test face validity and 50 participants in each country to test the internal consistency. The Cronbach's alpha value ranging from 0.86 to 0.97 indicated that the questionnaire has good internal consistency across all countries. It took approximately 8–10 mins to complete the survey.

### Data collection

As the researchers worldwide utilized social media platforms to collect data amid the global pandemic, a Google form survey link was distributed to online social media platforms (Facebook and WhatsApp) to recruit participants in this study. Participants were requested to pass on the questionnaire to their contacts or acquaintances in a pattern of snowball sampling. The outcomes of the study were, on each occasion, whether people believed or not: (1) in the effectiveness of the vaccine against COVID-19; (2) there is no need for vaccination for the post-infected individuals; and (3) there is no need for vaccination when all others are already vaccinated.

### Socio-demographic characteristics and medical history

The socio-demographic characteristics of the participants collected were age, gender, religion, education, marital status, smoking, residence, employment status, and income level. Besides, the use of social media, satisfaction with online information related to COVID-19 and vaccines, the experience of online searching COVID-19 and vaccine information, websites surfed, and trusted online information were also explored *via* Google form. In addition, participants were asked to report their medical history related to chronic conditions and the extent of health impairment. All of these were considered the predictor variables.

### Outcome variables

The outcome of the study was to understand the perception toward vaccine effectiveness to COVID-19 vaccination. To measure this, three questions were developed as outcome variables that were whether people agreed or not: (1) vaccine can control the viral spread; (2) post-COVID-19 patients must take the vaccine; and (3) there is no need for vaccination when the total population is vaccinated.

### Statistical analysis

Logistic regression models were performed to show the predictors for perception toward vaccine effectiveness. The adjusted odds ratio (AOR) was used to nullify the effects of the potential confounders. The variables were selected using the backward method depending on an extensive literature search and the principle of parsimony in selecting potential predictors. Relevant assumptions were made to ensure the goodness of fit of each model, the absence of any multi-collinearity, and the homogeneity of variance of the residuals.

## Results

### Demographic information

Table 1 provides the comparative description of participants' demographics based on rural and urban residential sites. A total of 5,673 participants responded to the study, the majority of whom were female (56%), from urban areas (68%), Islam (61%), with tertiary level of education (72%), had full-time employment (38%) and sufficient income (52%), but not suffering from chronic diseases (86%) and health impairments (80%). These variables were reported to differ significantly between rural and urban areas except gender.

**Table 1:** Comparative description of participants' demographics according to rural and urban areas.

**Characteristics**	**Overall (*n =* 5,673)**	**Rural (*n =* 1,804)**	**Urban (*n =* 3,869)**	***p*** **-value**
**Gender**				0.062
Female	3,181 (56%)	979 (54%)	2,202 (57%)	
Male	2,492 (44%)	825 (46%)	1,667 (43%)	
**Religion**				**<0.001**
Buddhism	482 (8.5%)	84 (4.7%)	398 (10%)	
Christianity	1,258 (22%)	667 (37%)	591 (15%)	
Hinduism	316 (5.6%)	10 (0.6%)	306 (7.9%)	
Islam	3,470 (61%)	1,001 (55%)	2,469 (64%)	
Other	147 (2.6%)	42 (2.3%)	105 (2.7%)	
**Age [Median(Q1, Q3)]**	25 (21, 39)	23 (21, 32)	27 (22, 42)	**<0.001**
**Education**				**<0.001**
No formal education	53 (0.9%)	24 (1.3%)	29 (0.7%)	
Primary	158 (2.8%)	74 (4.1%)	84 (2.2%)	
Secondary	1,387 (24%)	443 (25%)	944 (24%)	
Tertiary	4,075 (72%)	1,263 (70%)	2,812 (73%)	
**Employment**				**<0.001**
Employed full time	2,155 (38%)	526 (29%)	1,629 (42%)	
Employed part time	416 (7.3%)	185 (10%)	231 (6.0%)	
Looking for Job	256 (4.5%)	111 (6.2%)	145 (3.7%)	
Other	520 (9.2%)	211 (12%)	309 (8.0%)	
Retired	165 (2.9%)	28 (1.6%)	137 (3.5%)	
Student	906 (16%)	150 (8.3%)	756 (20%)	
Unemployed	1,255 (22%)	593 (33%)	662 (17%)	
**Income**				**<0.001**
Completely sufficient	894 (16%)	179 (9.9%)	715 (18%)	
Less sufficient	1,103 (19%)	451 (25%)	652 (17%)	
Not sufficient	704 (12%)	290 (16%)	414 (11%)	
Other	50 (0.9%)	30 (1.7%)	20 (0.5%)	
Sufficient	2,922 (52%)	854 (47%)	2,068 (53%)	
Chronic diseases^*^				**0.008**
No	4,906 (86%)	1,568 (87%)	3,338 (86%)	
Yes	717 (13%)	211 (12%)	506 (13%)	
Health impaired by Chronic disease^**^				**<0.001**
No	4,553 (80%)	1,259 (70%)	3,294 (85%)	
Yes	801 (14%)	418 (23%)	383 (9.9%)	
Extent of health impairment^***^				**<0.001**
Moderately impaired	926 (16%)	387 (21%)	539 (14%)	
Not at all	1,823 (32%)	332 (18%)	1,491 (39%)	
Severely impaired	357 (6.3%)	189 (10%)	168 (4.3%)	

* 50 patients (0.9%) of participants didn't tell whether they had chronic diseases or not.

** 319 patients (5.6 %) of participants didn't tell whether they had any health impairment due to this chronic disease or not.

*** 2,567 patients (45%) of participants didn't answer the question regarding the extent of health impairment.

### Participants' online activities related to COVID-19 and vaccine

Table 2 depicts participants' online activities regarding COVID-19 and vaccine information based on rural and urban sites. The majority of participants did not like to use social media (such as Facebook and YouTube) frequently (86%) but had trust in online information (78%) and mostly surfed the WHO website for COVID-related information (62%). However, this study reported that participants were neutral on COVID-19 information received through online platforms (33%), using other than the English language for online search (76%), experiencing difficulty in finding COVID-19-related information online (55%), and had not surfed different websites (59%) for the same. The study also determined that most participants had a good relationship with the lower socioeconomic group of people in the community (57%). The majority of the participants confirmed that they could post-effective online posts related to the COVID-19 vaccine, and they may share some private information on themselves or others intentionally or non-intentionally (58%). However, they found it difficult to formulate a question or express their thoughts and feelings about the COVID-19 vaccine (53%).

**Table 2:** Comparative description of participants' online activity related to COVID-19 and vaccine according to rural and urban areas.

**Characteristics**	**Overall (*n =* 5,673)**	**Rural (*n =* 1,804)**	**Urban (*n =* 3,869)**	***p*** **-value**
**Using social media**				>0.9
Frequent	801 (14%)	256 (14%)	545 (14%)	
Low	4,872 (86%)	1,548 (86%)	3,324 (86%)	
**Trust on online information**				**<0.001**
No	1,262 (22%)	349 (19%)	913 (24%)	
Yes	4,411 (78%)	1,455 (81%)	2,956 (76%)	
Satisfaction with online information related to COVID-19^*^				**<0.001**
Dissatisfied	469 (8.3%)	154 (8.5%)	315 (8.1%)	
Neutral	1,860 (33%)	561 (31%)	1,299 (34%)	
Satisfied	1,736 (31%)	610 (34%)	1,126 (29%)	
Very dissatisfied	361 (6.4%)	148 (8.2%)	213 (5.5%)	
Very satisfied	328 (5.8%)	102 (5.7%)	226 (5.8%)	
**Language used in searching information online**				**<0.001**
English	1,372 (24%)	557 (31%)	815 (21%)	
Not English	4,301 (76%)	1,247 (69%)	3,054 (79%)	
**Experience of searching COVID-19 information online**				**<0.001**
Difficult	3,126 (55%)	1,072 (59%)	2,054 (53%)	
Easy	2,547 (45%)	732 (41%)	1,815 (47%)	
**Surfing different websites for COVID-19 information**				0.8
No	3,324 (59%)	1,062 (59%)	2,262 (58%)	
Yes	2,349 (41%)	742 (41%)	1,607 (42%)	
**Surfing WHO website for COVID-19 information**				0.4
Frequently	3,543 (62%)	1,142 (63%)	2,401 (62%)	
Rarely	2,130 (38%)	662 (37%)	1,468 (38%)	
**Effectiveness of online posting**				**0.017**
No	2,369 (42%)	712 (39%)	1,657 (43%)	
Yes	3,304 (58%)	1,092 (61%)	2,212 (57%)	
**Ability to effectively express thoughts about vaccine through social media**				0.085
No	3,032 (53%)	934 (52%)	2,098 (54%)	
Yes	2,641 (47%)	870 (48%)	1,771 (46%)	
**Good relationship with**				**<0.001**
Lower socioeconomic group	2,125 (37%)	770 (43%)	1,355 (35%)	
Higher socioeconomic group	3,548 (63%)	1,034 (57%)	2,514 (65%)	

* 919 patients (16%) of participants didn't answer the question regarding online information satisfaction.

### Participants' residential information

**Figure 1:**
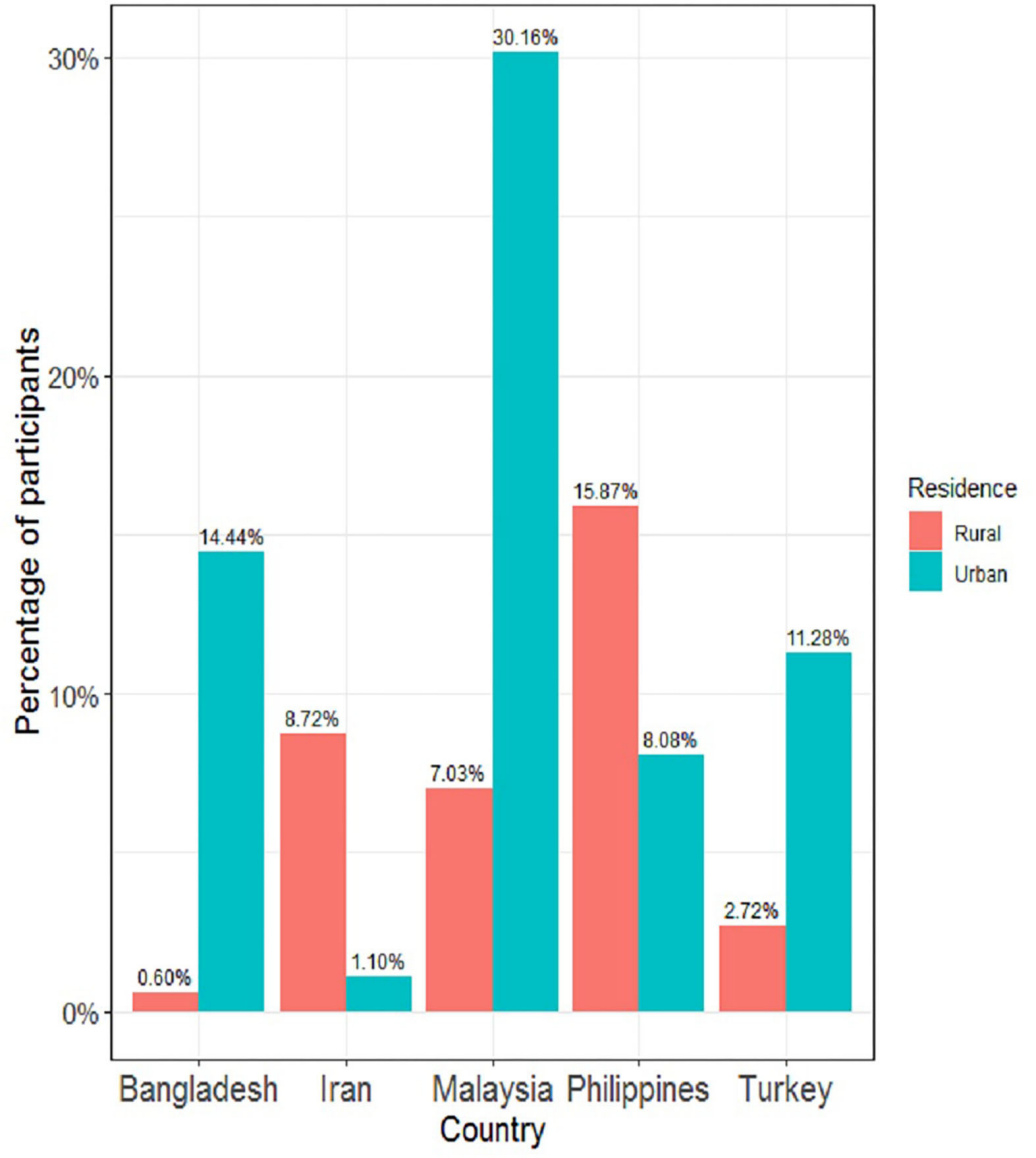
Country-wise distribution of participants in rural and urban area.

Figure 1 depicts the details of the top five countries of participants. Except for the Philippines and Iran, all three other countries' participants mostly lived in urban areas during data collection.

### Participants' response to COVID-19 vaccine

**Figure 2:**
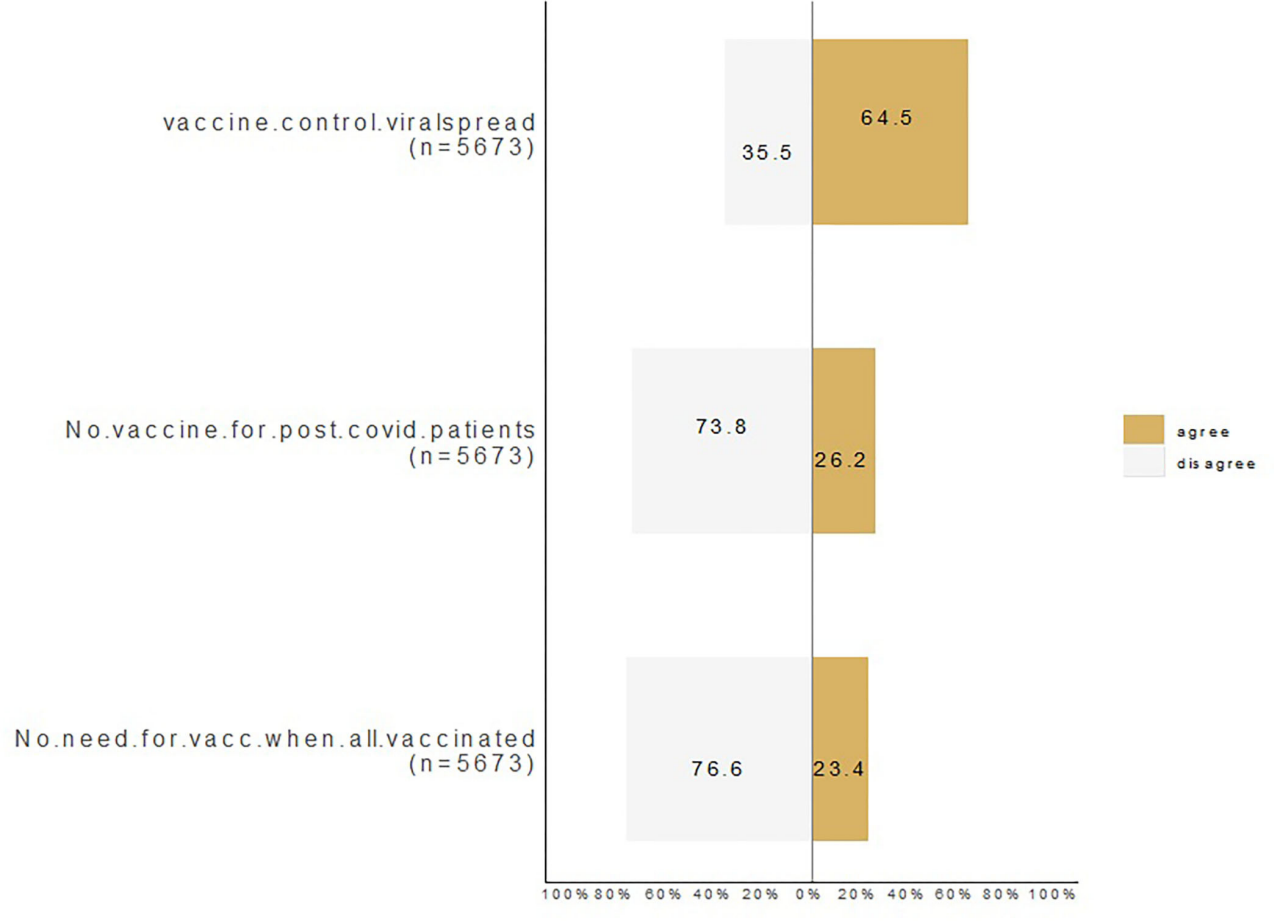
Participants' response on COVID-19 vaccine.

Figure 2 represents the distribution of the three primary outcomes of the study. Overall, 64% of participants agreed that the vaccine effectively controlled viral spread, 26% agreed that there was no need for vaccination for post-COVID-19 patients, and 23% agreed that there was no need for vaccination if others were vaccinated.

### Regression analysis between participants' variable and three main responses related to COVID-19 vaccine

Table 3 represents that participants' age, employment status, relationship with the different socioeconomic groups, income, experience of finding information online, surfing different websites, and trust in online information significantly affected their perception of vaccine effectiveness in controlling COVID-19 infection. Controlling all other variables, the study found that:

- Increasing age by 1 unit decreased the odds of trusting the vaccine's effectiveness by 4%.
- Students and retired participants had very high (2.07 and 1.81) odds of trusting the vaccine's effectiveness compared to all other participants' employment status, respectively.
- A good relationship with the socio-economically stable group has decreased the odds of trusting the vaccine's effectiveness by 32%.
- Sufficiency of income levels of the participants showed 22% lower odds on trust in vaccine effectiveness for controlling the infection.
- Participants' online information search related to COVID-19 exhibited 24% lower trust odds on the vaccine effectiveness.
- Participants who surfed different medical websites for COVID-related information had 35% higher trust odds on the vaccine effectiveness.
- Participants' trust in online information regarding COVID and vaccine information had 16% lower odds on trust in the vaccine effectiveness.

**Table 3:** Factors affecting the participants' agreement of vaccine effectiveness in controlling COVID-19 infection.

**Characteristics**	**AOR (95% CI)**		***p*** **-value**
**Age (in years)**	0.96 (0.95–0.97)		**<0.001**
**Employment**			
Unemployed	—		
Employed (Full time)	1.29 (1.09–1.54)		**0.004**
Employed (Part time)	1.46 (1.15–1.86)		**0.002**
Searching for employment	1.11 (0.83–1.48)		0.5
Other	0.94 (0.75–1.17)		0.6
Retired	1.81 (1.11–2.90)		**0.014**
Student	2.07 (1.72–2.49)		**<0.001**
**Good relationship with**			
Lower socioeconomic group	—		
Higher socioeconomic group	0.68 (0.60–0.77)		**<0.001**
**Income level**			
Not sufficient	—		
Completely sufficient	1.09 (0.87–1.37)		0.5
Less sufficient	0.88 (0.72–1.09)		0.2
Other	1.19 (0.65–2.18)		0.6
Sufficient	0.78 (0.64–0.94)		**0.010**
**Experience of searching COVID-19 information online**			
Difficult	—		
Easy	0.76 (0.67–0.86)		**<0.001**
**Surfing different websites for COVID-19 information**			
No	—		
Yes	1.35 (1.18–1.53)		**<0.001**
**Trust on online information**			
No	—		
Yes	0.84 (0.72–0.99)		**0.040**

AOR, Adjusted odds ratio; CI, Confidence interval.

Table 4 represents that participants' residential sites, gender, age, frequency of using social media, surfing different websites, including that of the WHO, for COVID-related information, participants' effective online posting, and their ability to express themselves online significantly affected their perception on no requirement of vaccination for post-COVID patients.

**Table 4:** Factors affecting the participants' agreement on no vaccination to post-COVID patients.

**Characteristics**	**AOR (95% CI)**		***p*** **-value**
**Residence**			
Rural			
Urban	1.34 (1.18–1.53)		**<0.001**
**Gender**			
Female			
Male	1.14 (1.01–1.29)		**0.041**
**Age (in years)**	0.99 (0.99–1.00)		**0.019**
**Using social media**			
Frequent			
Low	1.42 (1.18–1.71)		**<0.001**
**Effectiveness of online posting**			
No			
Yes	1.45 (1.29–1.66)		**<0.001**
**Ability to effectively express thoughts through social media**			
No			
Yes	1.18 (1.02–1.35)		**0.022**
**Surfing different websites for COVID-19 information**			
No			
Yes	1.22 (1.03–1.44)		**0.018**
**Surfing WHO websites for COVID-19 information**			
Frequently			
Rarely	0.73 (0.62–0.86)		**<0.001**

AOR, Adjusted odds ratio; CI, Confidence interval.

Adjusting all other variables' impacts, the study found that:

- Participants residing in urban areas had a 34% higher chance of believing that there was no need for vaccination for post-COVID-19 patients.
- Males had 14% higher odds of believing that there was no need for vaccination.
- Increasing age by 1 unit would decrease the chances of unbelief on vaccination need for post-COVID patients by 1%.
- Less social media app users had 42% higher odds of unbelief in need for vaccination for post-COVID-19 patients.
- Those who could post-effectively on social media had 45% higher odds of unbelief in need for vaccination for post-COVID-19 patients.
- Those who could express their feelings effectively online had 18% higher odds of unbelief in need for vaccination for post-COVID-19 patients.
- Occasional visitors of the WHO website had 27% lower odds of believing there was no need for vaccination for post-COVID-19 patients.
- Those who surfed different websites for COVID-19 information had 22% higher odds of developing unbelief toward the need for vaccination for post-COVID patients.

Table 5 provides the details of factors such as language, employment, frequency of using social media, surfing WHO websites for COVID-related information, and participants' effective online posting significantly affected the perception of no vaccination requirement for post-COVID patients. Adjusting all other variables, the study found that:

- Non-English language users had 29% higher odds of believing that they need no vaccination.
- Students, full-time workers, part-time workers, and retired participants had 4.16, 1.01, 1.15, and 1.37 times higher odds of believing they did not need to be vaccinated when all other people got vaccinated.
- Those who could post online effectively had 58% higher odds of developing the belief of no need for vaccination when all other people got vaccinated.
- Less social media users had 39% higher odds of developing the belief in no need for vaccination than all other people who got vaccinated.
- Occasional visitors of the WHO website showed 32% lower odds of believing that they had not been vaccinated when all other people got vaccinated.

**Table 5:** Factors affecting the participants' agreement on no need of vaccination in case others got vaccinated.

**Characteristics**	**AOR (95% CI)**		***p*** **-value**
**Language used in searching information online**			
English			
Not English	1.29 (1.10–1.51)		**0.002**
**Employment**			
Unemployed			
Employed (Full-time)	1.01 (0.86–1.19)		>0.9
Employed (Part-time)	1.15 (0.89–1.51)		0.3
Searching for employment	0.67 (0.50–0.90)		**0.007**
Other	0.75 (0.60–0.94)		**0.014**
Retired	1.37 (0.93–2.06)		0.12
Student	4.16 (3.11–5.64)		**<0.001**
**Effectiveness of online posting**			
No			
Yes	1.58 (1.37–1.82)		**<0.001**
**Using social media**			
Frequent			
Low	1.39 (1.15–1.68)		**<0.001**
**Surfing WHO website for COVID-19 information**			
Frequently			
Rarely	0.68 (0.59–0.79)		**<0.001**

AOR, Adjusted odds ratio; CI, Confidence interval.

## Discussion

The vaccination is the most appropriate approach for preventing and spreading COVID-19. However, peoples' perceptions toward vaccine effectiveness have fluctuated with the information flow on various social media channels and the severity of COVID cases (24, 25). Therefore, it is important that the current scenario of peoples' perception toward vaccine effectiveness and determinants affecting the same be explored to promote the vaccination approach against COVID-19 prevention and transmission effectively. This multinational study, highly representing the Asian countries, determined that nearly two-thirds of the public perceived the vaccine's effectiveness positively; however, nearly one in four people perceived that vaccination was not needed for post-COVID patients and that others were vaccinated. Haque et al. (6) reported that people with chronic diseases were less interested in vaccination in Bangladesh. The acceptance rate was higher among adults aged 30 years and above and among high-income groups (6). A systematic review carried out by Cascini et al. analyzed different countries' vaccine hesitancy profiles and found a fluctuating pattern of vaccine hesitancy, with an initial decrease followed by increased rates (4).

### Perception toward vaccine effectiveness in controlling COVID-19 spread

This large-scale multinational survey determined that more than half (64%) of participants agreed that vaccines effectively controlled COVID spread. Similarly, high vaccine acceptance was previously seen in the study of the United States (78%, 1,878 samples) conducted in June 2020, six sub-Saharan African countries (82.55%, 11,895 samples) conducted from October to December 2020, and a global survey encompassing 17 countries in the American, European, and Asian regions (90.4%, 19,714 samples) conducted in January to March 2021 (15, 25). The lower increment in vaccine hesitancy can be attributed to the attempt of countries on the strict vaccination campaigns with the certification before traveling and working globally, and the most appropriate reason experienced by the public was the absence of any other preventive alternatives over vaccines at the later phase. However, compared to similar studies, this study reported relatively higher hesitancy (15, 26, 27).

Aligning with our finding, the recent study conducted in Ethiopia showed hesitancy of vaccination by only half of the participants. Hence, it shows an incline trend to vaccine hesitancy over the period of time, so the appropriate awareness regarding vaccine effectiveness needs to be immediately provided. Further exploration determined that those who searched different websites for vaccine information, and students, retired, and working personnel had a higher positive perception of vaccine effectiveness. Vaccination has been made as a preliminary step for every public movement, work, and different activities that probably have encouraged people to accept it. However, increases in age, good relationships with higher socioeconomic groups, people having ease in finding vaccine-related information, and higher trust in online information had low odd value (<1) on vaccine effectiveness perception. This probably could reflect the trust of the elderly in biased, inappropriate, and fake information available on online platforms. In fact, the recent study also confirmed that people's vaccine acceptance or hesitancy was highly influenced by the information distributed in social media (24, 28).

Overall, it is clearly confirmed that public generally look social media and website for obtaining the true information, they need and get influenced by the information shared there. Hence, the concerned healthcare awareness organization and government should monitor and control to pass the genuine knowledge to public and change their perception and behavior accordingly. Similar to our finding, a recent review on determinants of COVID-19 vaccines in low- and middle-income countries (LMICs) also reported that occupation (specifically healthcare worker) and higher education had lower hesitancy of COVID-19 vaccines (29). Furthermore, recent reviews emphasized that improper awareness of public trust in vaccine effectiveness was the typical determinant of vaccine hesitancy (29). Similarly, previous data of the WHO/UNICEF showed that scientific evidence-based information, awareness, and knowledge, and cultural or socioeconomic parameters were the prominent factors affecting vaccine acceptability (30). In addition, Hassan et al. reported that the belief of COVID infection treatment by traditional method had 37% higher odds to develop vaccine hesitancy (28). On the contrary, social media and online information were reported to have a comparatively very high impact on public perception (31, 32). Therefore, proper orientation of the public toward utilizing the online platform, trustworthy resources for healthcare-related information, and proper dissemination of accurate information through the online portal conveniently are crucial to improve the public perception of current vaccination.

### Perception toward the need for vaccination for post-COVID patients

COVID-19 has been transmitted to a wide range of populations and countries. Although the infected participants may have developed immunity against the virus after an infection, timely vaccination has been considered appropriate and promoted (33). Conversely, this study determined that around one-fourth of the public (26%) still perceived no need for vaccination for post-COVID patients. Similarly, people living in urban places, male, less social media but high website users for COVID-19 information, and those who expressed their opinion effectively online had relatively higher odds of developing a perception of no need for vaccination for post-COVID patients. Participants living in urban places and surfing websites more for COVID information were naturally expected to have lower odds of having inappropriate perception; however, it was not found coherent. This probably has been the consequence of inappropriate availability and accessibility of accurate information related to COVID or the inability of the public to search and differentiate accurate information on COVID. A recent study of Ethiopia reported that people have a perception of further deterioration of their existing medical problem and even an understanding of suffering by COVID infection after COVID vaccination. Hence, the major concern toward the inappropriate perception existed for vaccination is the lack of unbiased information and awareness to the community. Therefore, the concerned authorities of the respective country must take appropriate action to facilitate the proper dissemination of scientific evidence-based information among the public through social media networking and government health-related websites. For instance, awareness campaigns *via* social media posting by the government of Macao were reported to influence significantly through higher patient engagement during the COVID-19 pandemic (34). Similarly, the active engagement of doctors and their recommendation to patient on vaccination have been reported to reduce hesitancy significantly in China (35). The Austrian study from King et al. displayed similar results and showed that doctor's recommendation greatly influences the decision-making process, and tailored vaccine information can support a higher vaccine coverage (36).

### Perception toward the need for vaccination if others were vaccinated

Lastly, this study found that more than three-fourths of people perceived no need for vaccination if others were vaccinated. It confirmed that people genuinely do not willing to get vaccinated. They do not have true faith in the safety and efficacy of vaccines, but rather, they were looking for another option of not getting vaccinated themselves. Also, the non-English users, students, and fewer social media users but with practical social media posting abilities had higher odds of having the perception of no need for vaccination in case others were vaccinated. Finding language as an associated factor in enhancing false perceptions toward COVID vaccines was also a prominent health-related error. This finding reflected a requirement to disseminate authentic information on COVID to students through understandable native languages, which could be non-English. For instance, a study on government social media engagement on Facebook during the COVID-19 pandemic in Macao reported a positive impact in attracting public engagement through the COVID-related information transmission *via* the government's official Facebook page. Interestingly, the personnel surfing the WHO websites for Covid information had an appropriate perception with lower odds (33). In addition, the information needed to be transmitted to attract, convince, and remove the misunderstanding to the listener rather than just sharing the information as a part of fulfilling the duty. People in Nigeria who do not have trust on the government have significantly reported to show higher hesitancy. Therefore, the confidence of the government and the information providing organization or media is another important factors that affected the people having hesitancy to COVID-19 vaccination.

### Strength and limitation

This multi-country survey is among a few studies exploring factors that may contribute to COVID-19 vaccine uptake improvement using extensive data collected from populations in countries with different socioeconomic and cultural contexts. However, this study has several limitations. Due to our study's cross-sectional nature, we cannot determine whether the outcome followed exposure or exposure followed exposure. Another limitation is the mode of study. Since we used a web-based self-administration mode of survey, there could be potential bias among the participants in responding to the survey questions. However, due to the restrictions related to the pandemic, this was the best mode currently available. Further studies are warranted to explore the relative importance of various vaccine-related, contextual, and individual or group determinants associated with the hesitancy of the COVID-19 vaccines. Moreover, analyzing the results from the missing 15 countries of the global survey and contrasting the outcomes with countries like Austria, Germany, Egypt, or Nigeria might give a broader insight due to cultural differences, social media usage, and urbanization rate. Given the exceptionally high burden of disease for COVID-19, urgent interventions and policies targeting the identified factors are necessary to decrease hesitancy for a COVID-19 vaccine. Targeting vaccine hesitancy is necessary to establish herd immunity worldwide and normalize life with COVID-19.

## Conclusion

This multinational online survey is among a few studies exploring factors that may contribute to the perception toward vaccine effectiveness in controlling COVID-19 spread in rural and urban communities in countries with different socioeconomic and cultural contexts. The vaccine is the most appropriate approach for preventing and spreading COVID-19. The perception toward vaccine effectiveness in controlling COVID-19 was greatly influenced by the social media information and by geography. The participants residing in urban areas had a higher chance of believing that there was no need for vaccination for post-COVID-19 patients.

Thus, governments need to raise awareness campaigns in rural areas. Doctor's recommendation and tailored vaccine information can support a higher vaccine coverage and influences the decision-making process. Individuals who gathered unfiltered information, surfed different websites, and consumed fake news for COVID-19 information generated a higher vaccine hesitancy toward the need for vaccination for post-COVID patients than visitors of the WHO website who had lower odds of believing there was now need for vaccination for post-COVID-19 patients. Society is realizing that social media has been deployed to increase social discord and decrease social cohesion. Fake news can be used to manipulate elections, health and vaccination programs, and lives. Awareness campaigns and policies need to be installed to diminish the damage from social media abuse. To promote vaccine acceptance, as experienced in Macao, the concerned authorities must provide the information in a most appropriate way to prevent confusion and misbelief and increase vaccine acceptance.

## Data availability statement

The raw data supporting the conclusions of this article will be made available by the authors, without undue reservation.

## Ethics statement

The study was approved by the Asia Metropolitan University Medical Research and Ethics (Ref. AMU FOM 0400132021). The patients/participants provided their written informed consent to participate in this study.

## Author contributions

All authors contributed equally.

## Conflict of interest

The authors declare that the research was conducted in the absence of any commercial or financial relationships that could be construed as a potential conflict of interest.

## Publisher's note

All claims expressed in this article are solely those of the authors and do not necessarily represent those of their affiliated organizations, or those of the publisher, the editors and the reviewers. Any product that may be evaluated in this article, or claim that may be made by its manufacturer, is not guaranteed or endorsed by the publisher.
